# Effectiveness and safety of dermal matrix used for diabetic foot ulcer: a systematic review and meta-analysis of randomized controlled trials

**DOI:** 10.1186/s12902-024-01550-3

**Published:** 2024-02-20

**Authors:** Lei Sui, Qiang Xie, Hong-tao Jiang, Xiao-dong Li

**Affiliations:** Department of Hand Foot Surgery, Affiliated Hospital of Chengde Medical University, No. 36 Nanyingzi Street, Shuangqiao District, Chengde City, 067000 Hebei Province China

**Keywords:** Dermal matrix, Diabetic foot ulceration, Wound healing, Meta-analysis

## Abstract

**Background:**

Diabetic foot ulcers (DFUs) have become a global health concern, which can lead to diabetic foot infection (DFI), lower leg amputation, and even mortality. Though the standard of care (SOC) practices have been recognized as the “gold standard” for DFU care, SOC alone may not be adequate to heal all DFUs and prevent their recurrence. The use of dermal matrix has emerged as an adjuvant treatment to enhance DFU healing. The current study aimed to evaluate the effectiveness and safety of dermal matrix application as an adjuvant treatment to the SOC.

**Methods:**

The databases of PubMed, Embase and CENTRAL were independently searched by two authors, with the following key terms: “diabetic foot ulcer”, “acellular dermal matrix”, “wound healing”, and so on. Randomized controlled trials (RCTs) evaluated the efficacy and safety of dermal matrix in the treatment of DFUs were eligible for inclusion. The primary outcomes analyzed included time to complete healing and complete healing rate at the final follow-up, while secondary outcomes included wound area, ulcer recurrence rate, amputation risk and complication risk. Meta-analyses were performed using random-effect or fixed-effect models, based on the heterogeneity test.

**Results:**

This study included a total of 15 RCTs with a total of 1524 subjects. Of these, 689 patients were treated with SOC alone, while 835 patients received SOC plus dermal matrix. Compared to the SOC group, significantly shorter time (MD = 2.84, 95%CI: 1.37 ~ 4.32, *p* < 0.001***) was required to achieve complete healing in dermal matrix group. Significantly higher complete healing rate (OR = 0.40, 95%CI: 0.33 ~ 0.49, *p* < 0.001***) and lower overall (RR = 1.83, 95%CI: 1.15 ~ 2.93, *p* = 0.011*) and major (RR = 2.64, 95%CI: 1.30 ~ 5.36, *p* = 0.007**) amputation risks were achieved in dermal matrix group compared to SOC group. No significant difference was found in the wound area, ulcer recurrence rate, and complication risk between the two groups.

**Conclusions:**

The application of dermal matrix as an adjuvant therapy in conjunction with SOC effectively improved the healing process of DFUs and reduced the amputation risk when compared to SOC alone. Furthermore, dermal matrix application was well tolerated by the subjects with no added complication risk.

**Supplementary Information:**

The online version contains supplementary material available at 10.1186/s12902-024-01550-3.

## Introduction

Diabetic foot ulcers (DFUs) have become a global health concern, with an estimated incidence of 19 to 35% in patients with diabetes mellitus [1]. It has been reported by the International Diabetes Federation that there will be 9.1–26.1 million patients develop DFUs annually [1]. Patients with DFUs are related with decreased quality of life (QoF) and increased risk of depression [2, 3]. Furthermore, diabetic foot infection (DFI) is more frequent in the DFU patients due to the incomplete skin and exposed bone, which may result in increased amputation risk to as high as 92% [4, 5]. It was reported that approximately 1 in 6 DFU patients will suffer from amputation, causing a mortality rate of about 47% within 5 years and a recurrence risk as high as 66% [6, 7].

DFUs treatment is associated with about 1/3 of the total diabetic care cost [8]. The primary goal of DFU treatment is to promote the re-epithelialisation of wound to reduce the complications risk associated with ulceration and to improve the patient’s QoF to a ‘pre-ulceration’ status. Besides glycemic control and revascularization, standard of care (SOC) treatment has been commonly selected as the conventional application for DFU wound management, which usually consists of the surgical sharp debridement, wound moist dressing, application of removable or irremovable off-loading device, and infection control [7, 9, 10]. The review of Everett et al. [11] summarized a total of 7 critical SOC practices, including surgical debridement, dressings promoting a moist wound environment, wound off-loading, vascular assessment, treatment of active infection, glycemic control, and ultidisciplinary care. Although these SOC practices are considered the “gold standard” for DFU care, the 20-week healing rate of DFU after SOC was less than 30% [12], and 40 and 65% of healed DFUs will recur within 1 year and 5 years, respectively [1]. Therefore, current SOC alone may not be sufficient to heal all DFUs and prevent their recurrence [13].

In recent years, a broad spectrum of novel treatments have been developed to improve diabetic wound healing. In areview by Snyder et al. [14], they identified a total if 76 commercially available skin substitutes used to treat chronic wounds. The majority of these substitutes do not contain cells and are derived from human placental membrane (the placenta’s inner layer), animal tissue, or donated human dermis allograft. These skin substitutes, whether allogeneic or xenogeneic graft, could provide the essential structure of extracellular matrix, signals for cellular migration, proliferation, angiogenesis, and endogenous matrix production and biochemical functions for enhancing wound healing [15, 16]. Many studies have demonstrated that these dermal matrices are effective when applied as adjuvant treatment to enhance DFUs healing [17–19]. However, high-level evidence to comprehensively illustrate the effectiveness and safety of SOC plus dermal matrix over SOC alone is still scarce.

Thus, the current systematic review will be conducted with the aim of evaluating the effectiveness and safety of dermal matrix application as an adjuvant treatment of SOC, basing on the available evidence from randomized controlled trials (RCTs).

## Materials and methods

This study was carried out in accordance with the PRISMA (Preferred Reporting Items for Systematic Reviews and Meta-Analyses) guideline [20], and the checklist is presented in Supplementary Appendix 1.

### Data sources

The following three databases were independently searched by two authors: PubMed, Embase and CENTRAL. The searching was completed using a method of combination of subject and free terms, with the following key terms: “diabetic foot ulcer”, “acellular dermal matrix”, “cellular dermal matrix”, “wound healing”, and so on. No restriction on the publication countries/ regions and publication date, while the publication language was restricted on English. Additionally, the references lists of the included studies were reviewed, and the potential related studies were hand searched and screened for eligibility.

### Inclusion and exclusion criteria

The retrieved records from the three databases were screened according to the following inclusion criteria: (1) patients: diagnosed with DFU; (2) intervention: biogenic skin substitutes for enhancing DFU healing, whether allogeneic or xenogeneic dermal matrix graft; (3) comparison: between SOC and dermal matrix; (4) outcomes: treatment outcomes of DFU, including ulcer healing rate, healing time, wound area, ulcer recurrence, amputation risk and complication risk; (5) studies: only prospectively designed RCTs were eligible.

Studies were excluded according to the following criteria: (1) patients with ulcers on foot caused by reasons other than diabetes, or patients with ulcers caused by diabetes on the lower leg; (2) patients treated with methods other than dermal matrix or SOC; (3) no available data on the effectiveness and safety outcomes; (4) studies designed as case series, cohort study, retrospective case-control study, systematic review/ meta analysis, literature review, and so on.

### Study screening and data extracting

Two authors independently screened all electronic records retrieved from the databases, according to the inclusion and exclusion criteria to select eligible studies. At the beginning, the records were imported into the EndNote software version X9 to eliminate the duplicates. Then, the two authors reviewed the titles/ abstracts of the remaining non-duplicates, to remove clearly irrelevant studies. After then, the full-text of the remained studies was downloaded and reviewed to evaluate the eligibility for inclusion.

The data extraction process was also completed by two authors independently, to obtain the following items: (1) study characteristics: the first author’s name, publication year, corresponding country/ region, study period, and follow-up time; (2) subjects characteristics: patients number, dropped patients number, male percentage, mean age, mean BMI, diabetes type (type I or type II), length of diabetes history, glycosylated hemoglobin percentage (HbA1c%), ankle-brachial index (ABI), patients with diabetic polyneuropathy (DPN), DFU grade according to Wagner or other classifications, DFU site (planta, dorsal, or other sites), DFU size, and DFU age; (3) treatment details: treatment regimens in screening phase and treatment phase, screening criterion for randomization in screening phase, and dermal matrix product; (4) outcome evaluations: ulcer healing rate, healing time, wound area, ulcer recurrence, amputation risk and complication risk.

### Quality assessment

The quality of the included RCTs was assessed using the Cochrane Collaboration tool for risk of bias assessment [21], which evaluates a total of 7 kinds of biases for RCTs as follows: (1) randomization sequence generation, (2) allocation concealment, (3) blinding of participants and personnel, (4) blinding of outcome assessment, (5) incomplete outcome data, (6) selective reporting and (7) other bias.

### Statistical analysis

When results of per-protocol (PP) and intention-to-treat (ITT) analyses were both reported, the ITT principle was followed in the analyses process. Comparisons of continuous outcomes (including time to complete heal and wound area, at final follow-up) between SOC and dermal matrix groups, were expressed as mean difference (MD) and 95% confidence interval (95% CI). Single-rate meta analysis was performed to calculate the pooled healing rate and ulceration recurrence rate of dermal matrix group at the final follow-up. Comparisons of dichotomous outcomes between SOC and dermal matrix groups, were expressed as odds ratio (OR, complete healing rate) and risk ratio (RR, ulcer recurrence rate, amputation risk and complication risk) as well as their 95% CIs. Heterogeneity among studies was estimated by I^2^ statistics. If I^2^ ≥ 50%, it indicates significant heterogeneity and random-effect model was applied. Otherwise, a fixed-effect model was used in case of non-significant heterogeneity.

If significant heterogeneity was detected, sensitivity analysis was performed by omitting each individual study sequentially to assess the impact of each study on the results. For outcomes reported in more than 5 studies, funnel plot was plotted, and publication bias was assessed using Egger’s and Begg’s tests (*p* < 0.100 and *p* < 0.050 were considered to indicate significant publication bias respectively). If significant publication bias was detected, non-parametric trim-and-filling method was used to adjust the publication bias. Data analyses were performed using the R language version 4.2.1 (R Foundation for Statistical Computing, Vienna, Austria). All statistical tests were two-sided and *P* value of less than 0.05 was considered significant.

## Results

### Study selecting

**Fig. 1:**
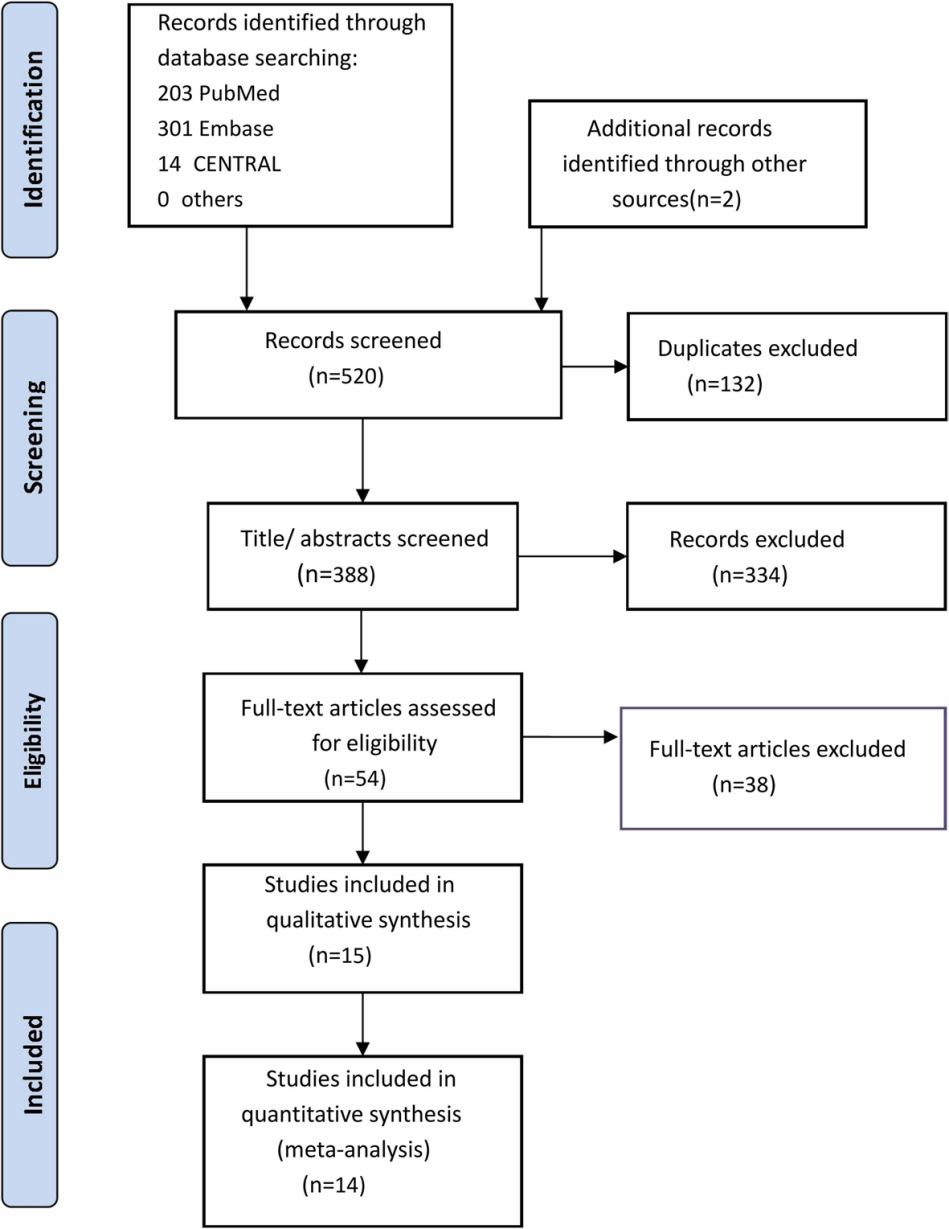
Flowchart of study searching and screening

The flow chart of studies screening is shown in Fig. 1. From the initial search, 520 studies were identified, of which 132 were duplicates that wereimmediately excluded. A futher 334 records were excluded after screening the titles/ abstracts, leaving 54 articles for full-text review. As a result, a total of 15 [22–36] and 14 [22, 23, 25–36] RCTs were included in the qualitative and quantitative synthesis (meta-analysis), respectively.

### Summary of the included studies

Table 1 summarizes the characteristics of the included studies. A total of 15 trials involving 1524 subjects were included in the analysis. The studies randomized a total of 689 patients to receive SOC alone and 835 patients to receive SOC plus dermal matrix. The male percentages were reported in 13 of the studies, ranging from 34.6 to 100.0%. The mean age was reported in 14 studies, ranging from 55.2 to 66.6 years. The mean BMI was available in 10 studies, ranging from 28.5 to 36.5 kg/m^2^. The follow-up periods were 4, 6, 12, 16, 21, 24, 28, and 42 weeks in 1 [22], 1 [36], 5 [25, 29–31, 34], 4 [23, 24, 27, 35], 1 [32], 1 [26], 1 [28] and 1 [33] studies, respectively. At the final follow-up, a total of 147 and 155 patients dropped out for follow-up.

**Table 1:** Characteristics of the included studies

Study ID	Study period	Country	Interventions	No. of patients	Male%	Mean age-years	Mean BMI	Diabetes type	Length of diabetes	Follow-up time	Dropped patients
Brigido, 2004 [22]	2003.4–2003.6	USA	SOC	20	77.5	Median: 58 (range: 43–70)	NA	NA	NA	weekly for 4 weeks	0
			GraftJacket tissue matrix	20			NA	NA	NA		0
Driver, 2015 [23]	2010.4–2013.11	USA	SOC	153	74.5	57.3 ± 9.7	34.1 ± 8.4	NA	NA	up to 16 weeks or until 100% wound closure	71
			SOC + IDRT	154	76.6	55.8 ± 10.6	34.0 ± 7.2	NA	NA		48
Cazzell, 2019 [24]	NA	USA	SOC + ADM allograft	61	75.4	55.2 ± 11.8	32.9 ± 7.4	I: 4.9%; II: 90.2%; Pre-D: 4.9%	NA	weekly for 16 weeks	14
Zelen, 2016 [25]	2014.12–2015.11	USA	SOC	20	60.0	57.1 ± 10.7	32.3 ± 6.9	NA	NA	weekly for 12 weeks	3
			SOC + human reticular CDM	20	80.0	61.5 ± 10.9	33.9 ± 8.7	NA	NA		1
Cazzell, 2017 [26]	NA	USA	SOC	69	73.9	56.9 ± 10.9	32.8 ± 6.9	I: 2.9%; II: 97.1%	NA	24 weeks with major endpoints at weeks 12, 16, and 24	13
			D-ADM	71	80.3	59.1 ± 12.8	32.6 ± 8.3	I: 5.6%; II: 90.1%	NA		18
			GJ-ADM	28	71.4	58.5 ± 9.8	31.4 ± 5.1	I: 7.1%; II: 92.9%	NA		5
Zelen, 2018 [27]	2014.12–2017.3	USA	SOC	40	70.0	59 .0 ± 12.0	35.0 ± 7.9	NA	NA	weekly for 16 weeks or until 100% wound closure	0
			SOC + human reticular CDM	40	60.0	62.0 ± 13.0	34.0 ± 8.8	NA	NA		0
Tchanque-Fossuo, 2019 [28]	2011.10–2016.8	USA	SOC	29	89.5	63.3 ± 9.1	36.5 ± 6.6	NA	NA	28w: 8-week treatment phase + 4-week maintenance phase + 4 monthly visits	8
			SOC + cellular Dermagraft	29	100.0	62.8 ± 9.0	32.4 ± 5.3	NA	NA		7
			SOC + acellular Oasis	31	94.7	61.9 ± 8.6	36.5 ± 11.6	NA	NA		9
Reyzelman, 2009 [29]	NA	USA	SOC	39	NA	58. 9 ± 11.6	34.6 ± 8.5	I: 5.1%; II: 94.9%	NA	up to 12 weeks or until 100% wound closure	2
			ADM	47	NA	55.4 ± 9.6	34.6 ± 6.7	I: 10.9%; II: 89.1%	NA		5
Hu, 2016 [30]	2010.9–2013.11	China	SOC + STSG	26	34.6	61.7 ± 12.1	NA	II: 100%	11.8 ± 7.8y	for 12 months after grafting surgery	2
			SOC + STSG + human ADM	26	42.3	66.6 ± 12.7	NA	II: 100%	15.0 ± 8.7y		1
Lantis, 2021 [31]	NA	USA	SOC	104	80.6	58.5 ± 11.9	32.2 ± 7.6	I: 6.7%; II: 93.3%	NA	weekly for 12 weeks or until 100% wound closure	22
			Graftskin	103	76.9	57.6 ± 11.5	33.3 ± 7.6	I: 10.7%; II: 89.3%	NA		24
Veves, 2001 [32]	NA	USA	SOC (saline-moistened gauze)	96	77.2	56.0 ± 10.0	33.1 ± 7.7	NA	NA	efficacy evaluation for 12 weeks + safety evaluation for another 3 months	22
			Graftskin	112	79.2	58.0 ± 10.0	30.9 ± 6.5	NA			22
Hahn, 2021 [33]	2016.4–2016.12	Korea	SOC (NPWT)	15	71.4	59.9 ± 13.4	NA	NA	16.3 ± 10.3y	every other day for 6 months or until 100% wound closure	0
			NPWT + micronized dermal matrix	15	73.3	63.5 ± 12.9	NA	NA	22.5 ± 14.7y		1
Cazzell, 2015 [34]	2013.5–2014.7	USA	SOC	41	73.0	56.6 ± 10.8	NA	NA	NA	up to 12 weeks or until 100% wound closure	4
			tri-layer porcine SIS	41	78.0	57.1 ± 10.9	NA	NA	NA		0
Brigido, 2006 [35]	NA	USA	SOC (sharp debridement)	14	NA	66.2 ± 4.4	NA	NA	NA	up to 16 weeks	0
			SOC + Graftjacket tissue matrix	14	NA	61.4 ± 7.2	NA	NA	NA		0
Campitiello, 2017 [36]	NA	Italy	SOC (wet dressing)	23	56.5	62.1 ± 7.7	28.9 ± 2.7	NA	NA	weekly for 6 weeks or until 100% wound closure	0
			Integra Flowable Wound Matrix	23	65.2	64.0 ± 8.9	28.5 ± 2.5	NA	NA		0

*SOC* standard of care, *IDRT* Integra Dermal Regeneration Template, *NA* not available, *ADM* acellular dermal matrix, *CDM* cellular dermal matrix, *D-ADM* DermACELL acellular dermal matrix, *GJ-ADM* GraftJacket acellular dermal matrix, *Pre-D* prediabetes, *STSG* split-thickness skin grafting, *NPWT* negative-pressure wound therapy, *SIS* small intestine submucosa, *BMI* body mass index

Table 2 summarizes the basic information about the status of diabetes mellitus and DFUs at randomization. HbA1c% levels were reported in 12 studies, with a range of 7.11 to 10.2%. The mean ABI was reported in 4 studies, with a range of 0.7 to 1.2. A total of 12 studies reported the mean DFU size, with a range of 1.3 to 32.1 cm^2^. The treatment details of the included studies are listed in Table 3. Before the treatment phase, 8 studies [23–25, 27, 28, 31, 32, 34] included screening phase and applied SOC treatment (surgical debridement, wound dressings, wound off-loading, and infection management) to the enrolled patients lasting for 1 or 2 weeks. At the end of the screening phase, the including criterion for randomization was set as less than 20% [25, 27], 30% [23, 31, 32] or 40% [28] reduction of wound area in the ulcer site. Many different dermal matrix products were applied for wound repair, with the GraftJacket matrix (Wright Medical Technology, Inc., Arlington, TN, USA) being the most commonly researched product in 4 RCTs [22, 26, 29, 35].

**Table 2:** Baseline information about the statuses of diabetes mellitus and diabetic foot ulceration (DFU)

Study ID	Interventions	No. of patients	HbA1c%	ABI	DPN%	DFU grade	Site of DFU	DFU size (cm^2^)	DFU age
Brigido, 2004 [22]	SOC	20	NA	NA	NA	NA	NA	NA	Mean: 25w
	GraftJacket tissue matrix	20							Mean: 27w
Driver, 2015 [23]	SOC	153	8.2 ± 1.9	NA	NA	Wagner 2:116 (75.8%)	Dorsal: 127(83.6%); Plantar: 25(16.5%)	3.7 ± 2.7	303 ± 418d
	SOC + IDRT	154	8.0 ± 1.8			Wagner 2: 109 (70.8%)	Dorsal: 126(81.8%); Plantar: 28(18.2%)	3.5 ± 2.5	308 ± 491d
Cazzell, 2019 [24]	SOC + ADM allograft	61	NA	NA	42.6%	Wagner 3: 59 (96.7%); Wagner 4: 2 (3.3%)	Ankle: 1(1.6%); Dorsal: 33(54.1%); Plantar: 26(42.6%); Plantar/dorsal: 1(1.6%)	29.0 ± 21.0	3.8 ± 3.4 m
Zelen, 2016 [25]	SOC	20	7.8 ± 1.8	NA	NA	NA	Toe: 7(35.0%); Forefoot: 7(35.0%); Midfoot: 2(10.0%); Ankle/hindfoot: 4(20.0%)	2.7 ± 2.3	NA
	SOC + human reticular CDM	20	7.9 ± 1.6				Toe: 6(30.0%); Forefoot: 5(25.0%); Midfoot: 7(35.0%); Ankle/hindfoot: 2(10.0%)	4.7 ± 5.2	
Cazzell, 2017 [26]	SOC	69	8.4 ± 1.9	NA	NA	Wagner 1: 14 (20.3%) Wagner 2: 55 (79.7%)	Dorsal: 15(21.7%); Plantar: 52(75.4%); Other: 2(2.9%)	3.6 ± 3.6	36.4 ± 36.4w
	D-ADM	71	8.5 ± 1.8			Wagner 1: 12 (16.9%) Wagner 2: 59 (83.1%)	Dorsal: 12(16.9%); Plantar: 56(78.9%); Other: 3(4.2%)	3.9 ± 4.2	40.0 ± 36.4w
	GJ-ADM	28	7.6 ± 1.4			Wagner 1: 5 (17.9%) Wagner 2: 23 (82.1%)	Dorsal: 6(21.4%); Plantar: 21(75.0%); Other: 1(3.6%)	3.3 ± 2.7	36.8 ± 53.6w
Zelen, 2018 [27]	SOC	40	7.8 ± 1.5	NA	100%	NA	Toe: 11 (28%); Forefoot: 18 (45%); Midfoot: 8 (20%); Ankle/hindfoot: 3 (7%)	3.2 ± 4.0	> = 4w
	SOC + human reticular CDM	40	7.6 ± 1.4				Toe: 13 (33%); Forefoot: 13 (33%); Midfoot: 6 (15%); Ankle/hindfoot: 8 (20%)	2.7 ± 2.4	
Tchanque-Fossuo, 2019 [28]	SOC	29	8.6 ± 1.7	1.07 ± 0.14	NA	NA	Dorsal: 3 (15.8%); Plantar: 15 (79.0%); Lateral: 1 (5.3%)	1.3 ± 0.9	21.7 ± 36.1w
	SOC + cellular Dermagraft	29	7.6 ± 1.5	1.22 ± 0.17			Dorsal: 1 (5.9%); Plantar: 13 (76.5%); Lateral: 2 (11.8%); Medial: 1 (5.9%)	1.6 ± 1.8	37.6 ± 96.1w
	SOC + acellular Oasis	31	7.7 ± 1.5	1.10 ± 0.12			Dorsal: 3 (15.8%); Plantar: 15 (79.0%); Lateral: 1 (5.3%)	3.1 ± 3.8	10.9 ± 7.6w
Reyzelman, 2009 [29]	SOC	39	7.6 ± 1.6	ranging from 0.7 to 1.2	NA	University of Texas (UT) grade 1 or 2	Toe: 5(12.8%); Foot: 17(43.6%); Heel: 8(20.5%); Other: 3(7.7%)	5.1 ± 4.8	22.9 ± 29.8w
	ADM	47	8.2 ± 2.0				Toe: 15(32.6%); Foot: 15(32.6%); Heel: 4(8.7%); Other: 5(10.9%)	3.6 ± 4.3	23.3 ± 22.4w
Hu, 2016 [30]	SOC + STSG	26	10.2 ± 1.1	0.9 ± 0.2	NA	Wagner grade 2 or 3	Ankle: 4 (15.4%); Dorsal: 6 (23.1%); Plantar: 7 (26.9%); Forefoot: 5 (19.2%); Heel: 4 (15.4%)	28.6 ± 25.2	25.0 ± 33.9w
	SOC + STSG + human ADM	26	9.8 ± 1.5	1.0 ± 0.2			Ankle: 6 (23.1%); Dorsal: 7 (26.9%); Plantar: 7 (26.9%); Forefoot: 2 (7.7%); Heel: 4 (15.4%)	32.1 ± 22.2	29.4 ± 41.7w
Lantis, 2021 [31]	SOC	104	8.3 ± 1.8	NA	59.6%	NA	Dorsal: 24 (23.1%); Plantar: 80 (76.9%)	3.8 ± 2.8	233.1 ± 312.9d
	Graftskin	103	8.1 ± 1.9		50.5%		Dorsal: 25 (24.2%); Plantar: 78 (75.8%)	3.6 ± 2.5	263.9 ± 514.5d
Veves, 2001 [32]	SOC (saline-moistened gauze)	96	8.6 ± 1.4	0.65–0.80: 10 (10.4%); 0.80–1.00: 29 (30.2%); > 1.00: 54 (56.3%)	100%	NA	Plantar: 100%	2.8 ± 2.5	11.1 ± 12.5 m
	Graftskin	112	8.6 ± 1.5	0.65–0.80: 10 (8.9%); 0.80–1.00: 50 (35.7%); > 1.00: 59 (52.7%)				3.0 ± 3.1	11.5 ± 13.3 m
Hahn, 2021 [33]	SOC (NPWT)	15	8.2 ± 2.4	0.8 ± 0.7	50.0%	Wagner grade 2 or higher	Ankle: 2(14.3%); Dorsal: 4 (28.6%); Plantar: 2 (14.3%); Forefoot: 3 (21.4%); Heel: 3 (21.4%)	13.1 ± 22.2	1–3 m: 5; 3-6 m: 7; > 6 m: 2
	NPWT + micronized dermal matrix	15	7.1 ± 1.8	0.7 ± 0.6	60.0%		Ankle: 3 (20.0%); Dorsal: 2 (13.3%); Plantar: 3 (20.0%); Forefoot: 4 (26.7%); Heel: 3 (20.0%)	16.3 ± 10.3	1–3 m: 4; 3-6 m: 8; > 6 m: 3
Cazzell, 2015 [34]	SOC	41	NA	NA	100%	NA	Plantar: 100%	2.6 ± 7.5	22.2 ± 13.5w
	tri-layer porcine SIS	41	NA					2.1 ± 2.3	21.3 ± 12.3w
Brigido, 2006 [35]	SOC (sharp debridement)	14	7.9 ± 0.6	NA	NA	Wagner grade 2	Plantar: 4(28.6%); Dorsal: 3(21.4%); Medial: 2(14.3%); Lateral: 3(21.4%); Other: 2(14.3%)	NA	NA
	SOC + Graftjacket tissue matrix	14	8.1 ± 1.0				Plantar: 5(35.7%); Dorsal: 3(21.4%); Medial: 5(35.7%); Other: 1(7.1%)		
Campitiello, 2017 [36]	SOC (wet dressing)	23	7.8 ± 0.8	Right: 0.94 ± 0.1; Left: 0.93 ± 0.1	NA	Wagner grade 3	Abscesses foot: 16 (69.6%); Heel: 2 (8.7%); Metatarsal head: 5 (21.7%)	NA	39.5 ± 9.9w
	Integra Flowable Wound Matrix	23	7.9 ± 0.8	Right: 0.92 ± 0.1 Left: 0.92 ± 0.1			Abscesses foot: 18 (78.3%); Heel: 1 (4.4%); Metatarsal head: 4 (17.4%)		38.56 ± 12.6w

*SOC* standard of care, *IDRT* Integra Dermal Regeneration Template, *NA* not available, *ADM* acellular dermal matrix, *CDM* cellular dermal matrix, *D-ADM* DermACELL acellular dermal matrix, *GJ-ADM* GraftJacket acellular dermal matrix, *STSG* split-thickness skin grafting, *NPWT* negative-pressure wound therapy, *SIS* small intestine submucosa, *ABI* ankle-brachial index, *DPN* diabetic polyneuropathy

**Table 3:** Treatment details of the included studies

Study ID	Interventions	Screening phase		Treatment phase	
		Treatment regimen	screening criterion	Intervention regimen	Dermal matrix product
Brigido, 2004 [22]	SOC	No screening phase		sharp debridement, wound gel with gauze dressings, offloading	Craftjacket tissue matrix (Wright Medical Technology, Inc., Arlington, Tenn)
	GraftJacket tissue matrix			surgical application of scaffold at day 0	
Driver, 2015 [23]	SOC	1) period: 2w; 2) treatment: infection and exudate management, sharp debridement, moist wound therapy, offloading device	≤30% reduction in ulcer site	sharp debridement, moist wound therapy, offloading	Integra Dermal Regeneration Template (IDRT; marketed as Omnigraft Dermal Regeneration Matrix)
	SOC + IDRT			IDRT was applied to the debrided wound, trimmed to size and secured with sutures or staples	
Cazzell, 2019 [24]	SOC + ADM allograft	1) period: 1w; 2) treatment: surgical debridement, offloading device, NPWT	none	surgical debridement, offloading, NPWT, Meshed 4*4 cm and 5*7 cm D-ADM were attached onto wound	DermACELL (D-ADM; LifeNet Health, Virginia Beach, Virginia)
Zelen, 2016 [25]	SOC	1) period: 2w; 2) treatment: surgical debridement, wound dressing, offloading device	≤20% reduction in ulcer site	surgical debridement, offloading, wound dressing	AlloPatch® Pliable™ (Musculoskeletal Transplant Foundation, Edison, NJ, USA)
	SOC + human reticular CDM			HR-ADM provided in size-specific grafts as small as 1.5*1.5 cm	
Cazzell, 2017 [26]	SOC	No screening phase		sharp debridement, moist wound treatment, off-loading	–
	D-ADM			meshed 4 × 4 cm (thickness: 0.5–1.0 mm) D-ADM	DermACELL; LifeNet Health, Virginia Beach, VA
	GJ-ADM			meshed 4 × 4 cm (thickness: 0.38–1.02 mm) GJ-ADM	GraftJacket; Wright Medical Technology, Memphis, TN
Zelen, 2018 [27]	SOC	1) period: 2w; 2) treatment: sharp debridement, wound dressing, offloading device	≤20% reduction in ulcer site	wound dressing, offloading	AlloPatch Pliable (MTF, Musculoskeletal Transplant Foundation, Edison, New Jersey)
	SOC + human reticular CDM			provided in sizes as small as 1.5 × 1.5 cm to optimize donor tissue	
Tchanque-Fossuo, 2019 [28]	SOC	1) period: 2w; 2) treatment: debridement, SOC dressing, offloading device, infection management	≤40% reduction in ulcer site	SOC wound dressing	–
	SOC + cellular Dermagraft			NA	Dermagraft (bioengineered ECM containing living fibroblasts)
	SOC + acellular Oasis			NA	Oasis (ECM devoid of living cells)
Reyzelman, 2009 [29]	SOC	No screening phase		moist-wound therapy, debridement, offloading	GraftJacket Regenerative Tissue Matrix – Ulcer Repair; Wright Medical Technology, Inc., Arlington, TN
	ADM			application of a human ADM (4*4 cm)	
Hu, 2016 [30]	SOC + STSG	No screening phase		patient education, glucose control, off-loading, wound moist treatment, debridement, infection control	human allograft ADM purchased from Jie-Ya Life Tissue Engineering (Beijing, China)
	SOC + STSG + human ADM			ADM was implanted onto the debrided wounds as a scaffold and covering	
Lantis, 2021 [31]	SOC	1) period: 2w; 2) treatment: sharp debridement, infection elimination, moist wound dressing, offloading device, infection management	≤30% reduction in ulcer site	sharp debridement, infection elimination, moist wound dressing, offloading device, infection management	fetal bovine dermis (FBADM) (PriMatrix, Integra LifeSciences, Princeton, US)
	Graftskin			meshed 4 × 4 cm graft placed in direct contact with a freshly debrided wound	
Veves, 2001 [32]	SOC (saline-moistened gauze)	1) period: 1w; 2) treatment: aggressive debridement, saline-moistened gauze	≤30% reduction in ulcer site	moist-wound therapy, off-loading	Graftskin (Apligraf; Organogenesis, Canton, MA, and Novartis Pharmaceuticals, East Hanover, NJ)
	Graftskin			Graftskin was placed directly over the ulcer site	
Hahn, 2021 [33]	SOC (NPWT)	No screening phase		surgical debridement, NPWT	MHADM (CG-PASTE; CGBio Co Ltd)
	NPWT + micronized dermal matrix			MHADM was molded to fit three-dimensional shape of wound	
Cazzell, 2015 [34]	SOC	1) period: 1w; 2) treatment: debridement, callus resection, offloading device	none	wound care/dressings	tri-layer porcine SIS (OASISUltra; Cook Biotech, Inc., West Lafayette, IN; exclusively marketed by Smith and Nephew, Inc., Fort Worth, TX)
	tri-layer porcine SIS			SIS was applied once each week to ulcers. The matrix was cut so that there was an approximate 1/8 in. overlap on the wound edge	
Brigido, 2006 [35]	SOC (sharp debridement)	No screening phase		sharp debridement, off-loading, wound dressing	Graftjacket (Wright Medical Technology, Inc., Arlington, TN, USA)
	SOC + Graftjacket tissue matrix			GraftJacket was prepared for implantation	
Campitiello, 2017 [36]	SOC (wet dressing)	No screening phase		sharp debridement, removable off-loading, wound dressing	Integra Flowable Wound Matrix, Integra LifeScience Corp, Plainsboro, NJ, USA
	Integra Flowable Wound Matrix			the matrix Integra TM Flowable Wound Matrix was applied to the lesion, using the flexible injector	

*SOC* standard of care, *IDRT* Integra Dermal Regeneration Template, *NA* not available, *ADM* acellular dermal matrix, *CDM* cellular dermal matrix, *D-ADM* DermACELL acellular dermal matrix, *GJ-ADM* GraftJacket acellular dermal matrix, *STSG* split-thickness skin grafting, *NPWT* negative-pressure wound therapy, *SIS* small intestine submucosa

**Fig. 2:**
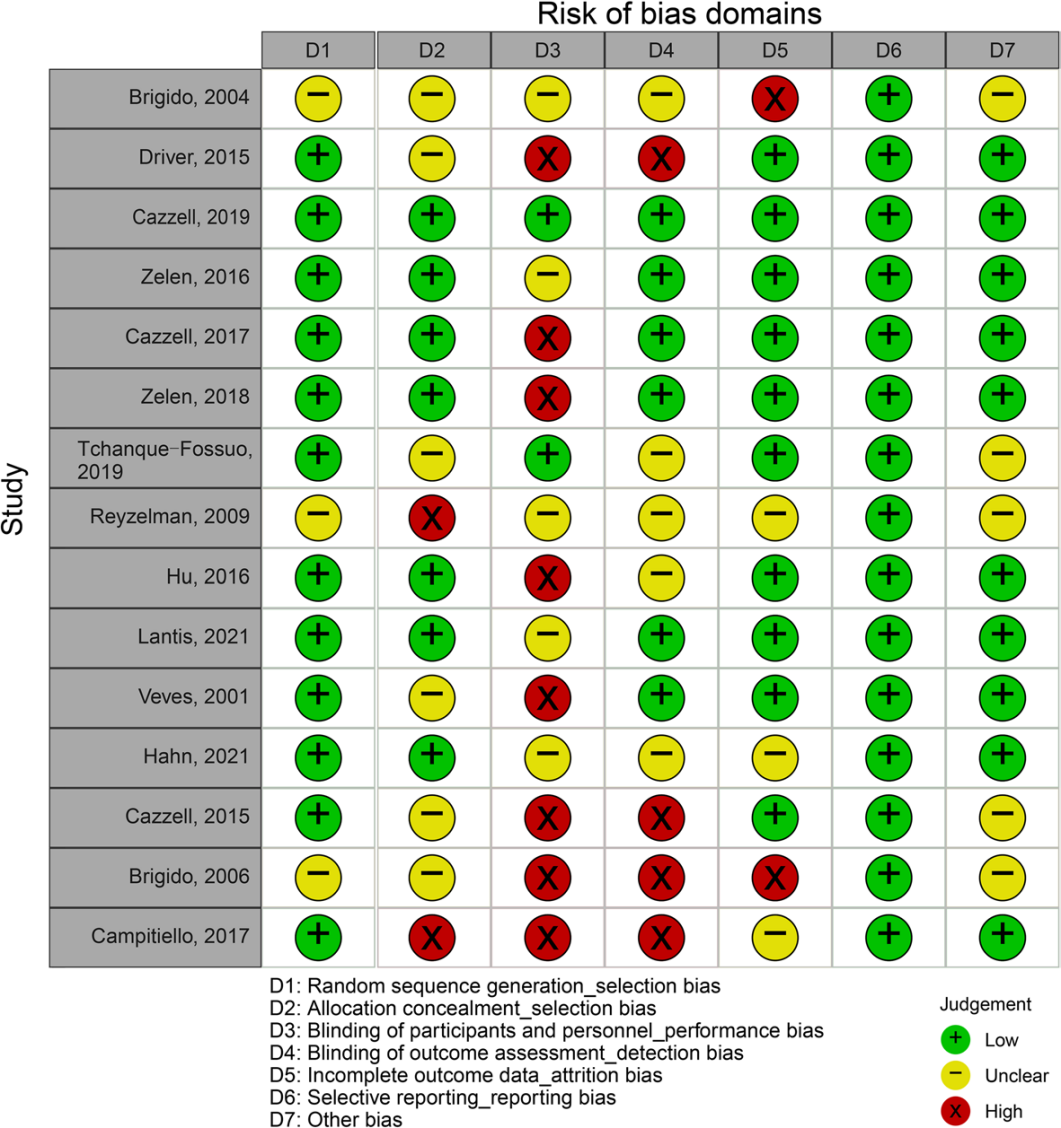
The light bulb diagram for quality assessment of the included studies

Figure 2 shows the results of the quality assessment of the included RCTs. Due to the obviously different treatment process, the blinding of the patients was difficult. As a result, there is a high risk of bias in the item of “blinding of participants and personnel”. The item “blinding of outcome assessment” was also presented with high risk of bias in 4 of the studies. The other items were of relatively low risk of bias.

### Effectiveness of the dermal matrix in DFU

**Fig. 3:**
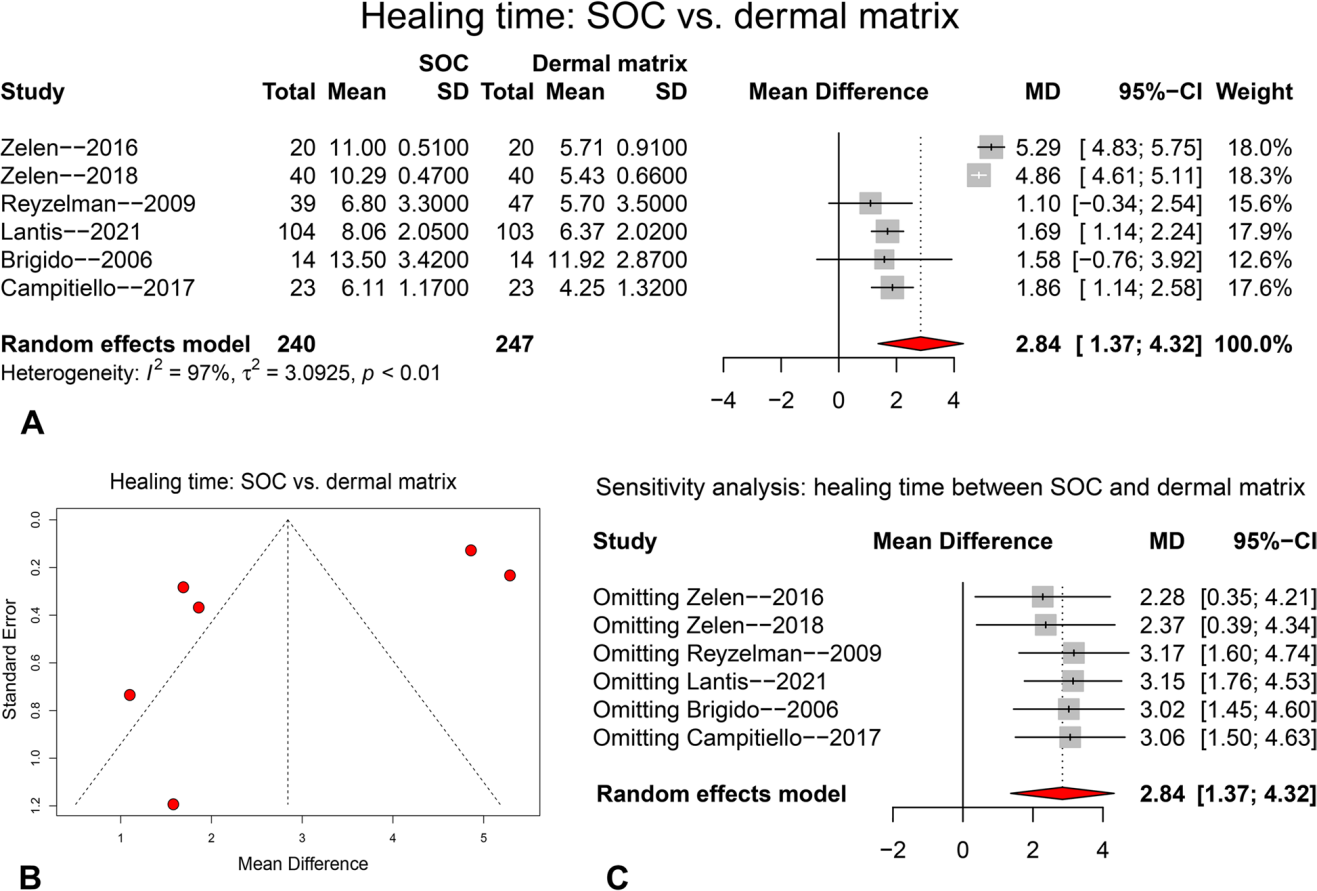
The pooling result for complete healing time compared between SOC and dermal matrix groups at final follow-up. **A** forest plot; **B** funnel plot; **C** forest plot for sensitivity analysis

The comparison of mean time to complete healing between SOC and dermal matrix is presented in Fig. 3. Six primary trials were pooled with random-effect model (I^2^ = 97%), and significantly shorter time was required to achieve complete healing in dermal matrix group compared to the SOC group (see the forest plot in Fig. 3A, MD = 2.84, 95%CI: 1.37 ~ 4.32, *p* < 0.001***). The funnel plot (Fig. 3B), Egger’s test (*p* = 0.143) and Begg’s test (*p* = 0.573) indicate that there is no significant publication bias. The forest plot of sensitivity analysis (Fig. 3C) showed there was no single study that significantly influenced the pooling result.

**Fig. 4:**
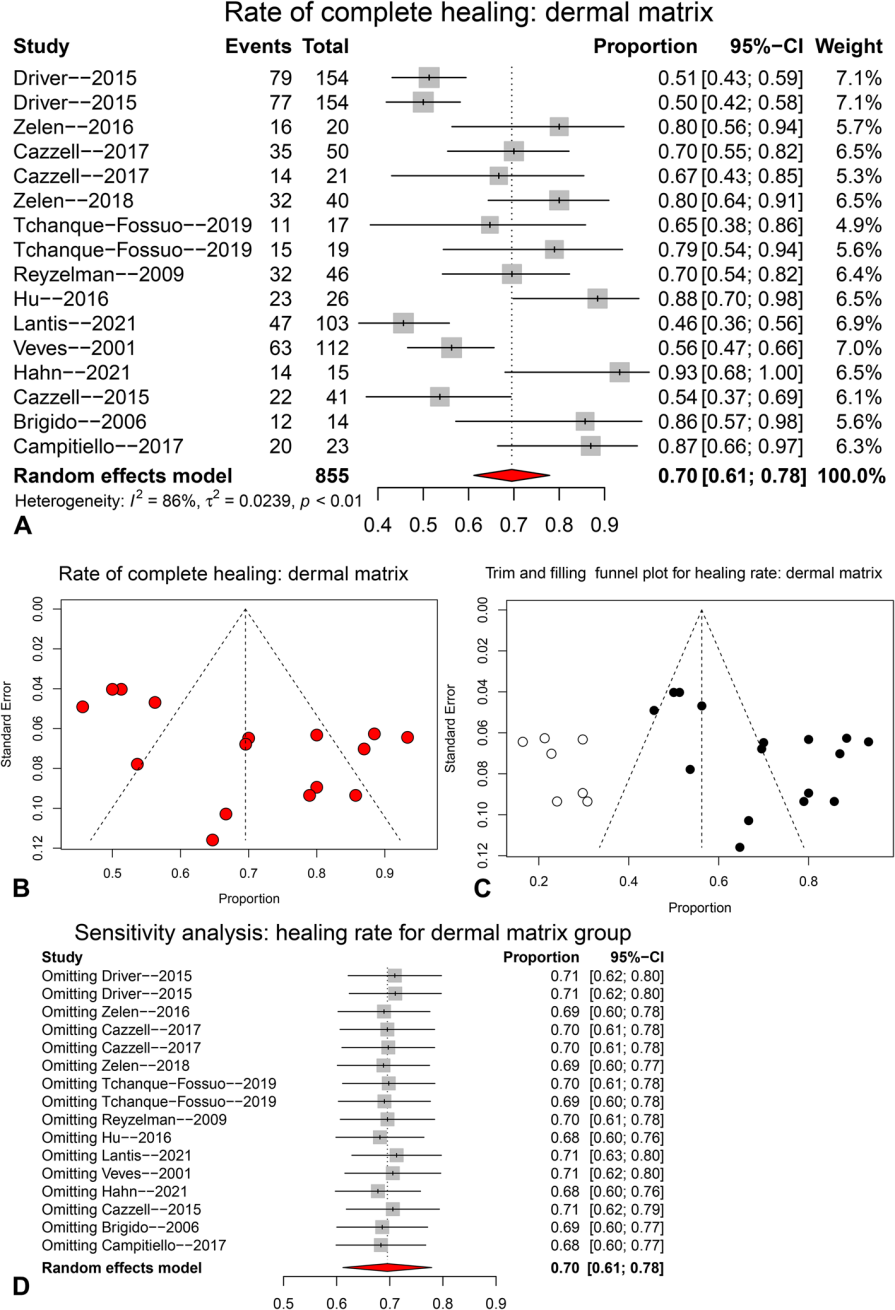
The pooling result for complete healing rate of dermal matrix group at final follow-up. **A** forest plot; **B** funnel plot; **C** trim and filling funnel plot; **D** forest plot for sensitivity analysis

The pooling result for complete healing rate of dermal matrix group at final follow-up is shown in Fig. 4. Thirteen studies and 16 arms were pooled with random effects model (I^2^ = 86%), and the pooled healing rate of dermal matrix group was 0.70 (95CI: 0.61 ~ 0.78) (see the forest plot in Fig. 4A). The forest plot of sensitivity analysis (Fig. 4D) revealed that none of the studies had a significant impact on the pooled result.. However, the funnel plot (Fig. 4B), Egger’s test (*p* = 0.012) and Begg’s test (*p* = 0.529) indicated the presence of significant publication bias. Therefore, a trim and filling funnel plot was generated, which resulted in an adjusted healing rate of 0.56 (95CI: 0.47 ~ 0.66) for the dermal matrix group (Fig. 4C).

**Fig. 5:**
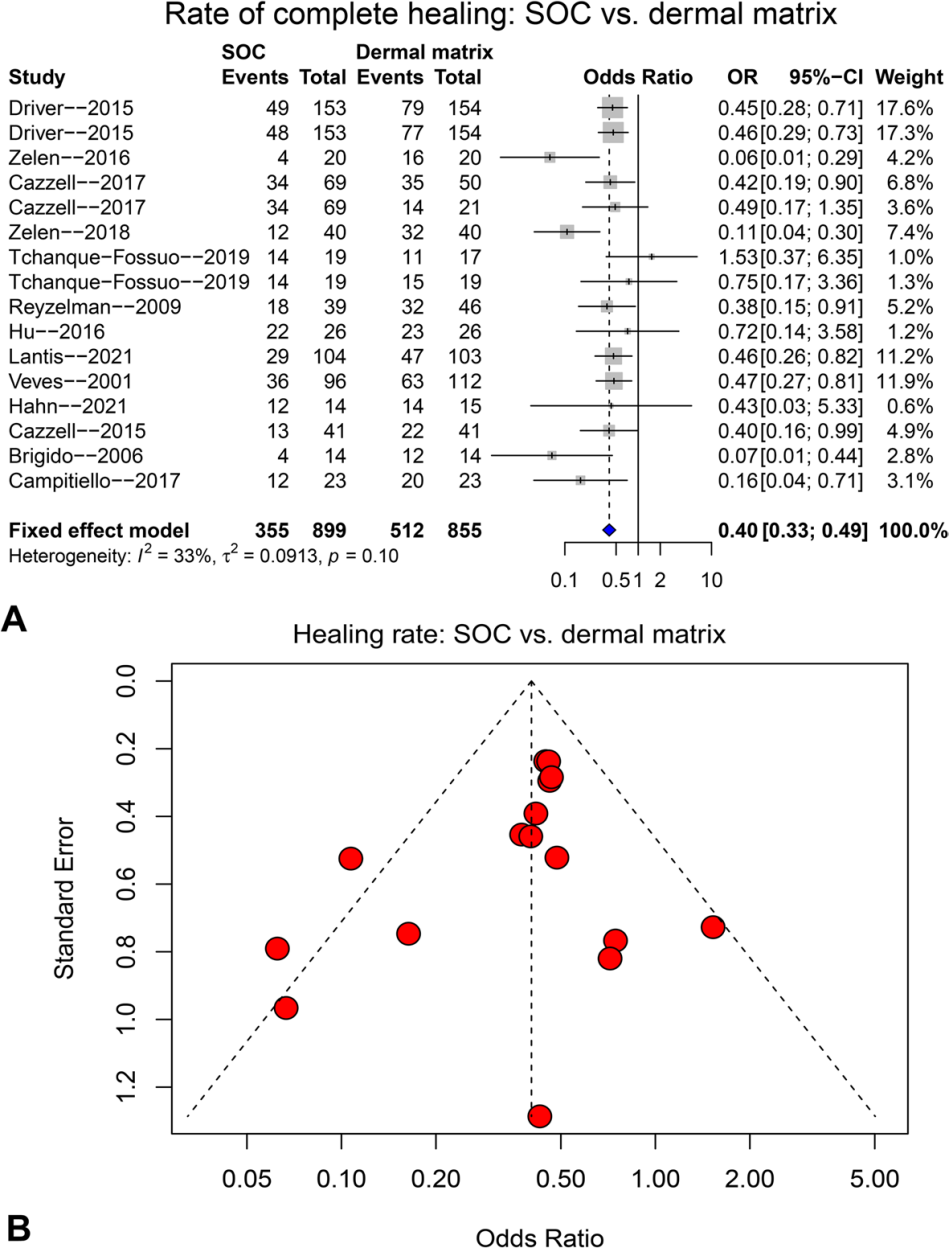
The pooling result for complete healing rate compared between SOC and dermal matrix groups at final follow-up. **A** forest plot; **B** funnel plot

The comparison of the complete healing rate between SOC and dermal matrix is presented in Fig. 5. Thirteen studies and 16 arms were pooled with fixed-effect model (I^2^ = 33%), resulting in a significantly higher complete healing rate in dermal matrix group compared to the SOC group (see the forest plot in Fig. 5A, OR = 0.40, 95%CI: 0.33 ~ 0.49, *p* < 0.001***). The funnel plot (Fig. 5B), Egger’s test (*p* = 0.224) and Begg’s test (*p* = 0.242) did not show any significant publication bias. Sensitivity analysis was not conducted as there was no significant heterogeneity.

**Fig. 6:**
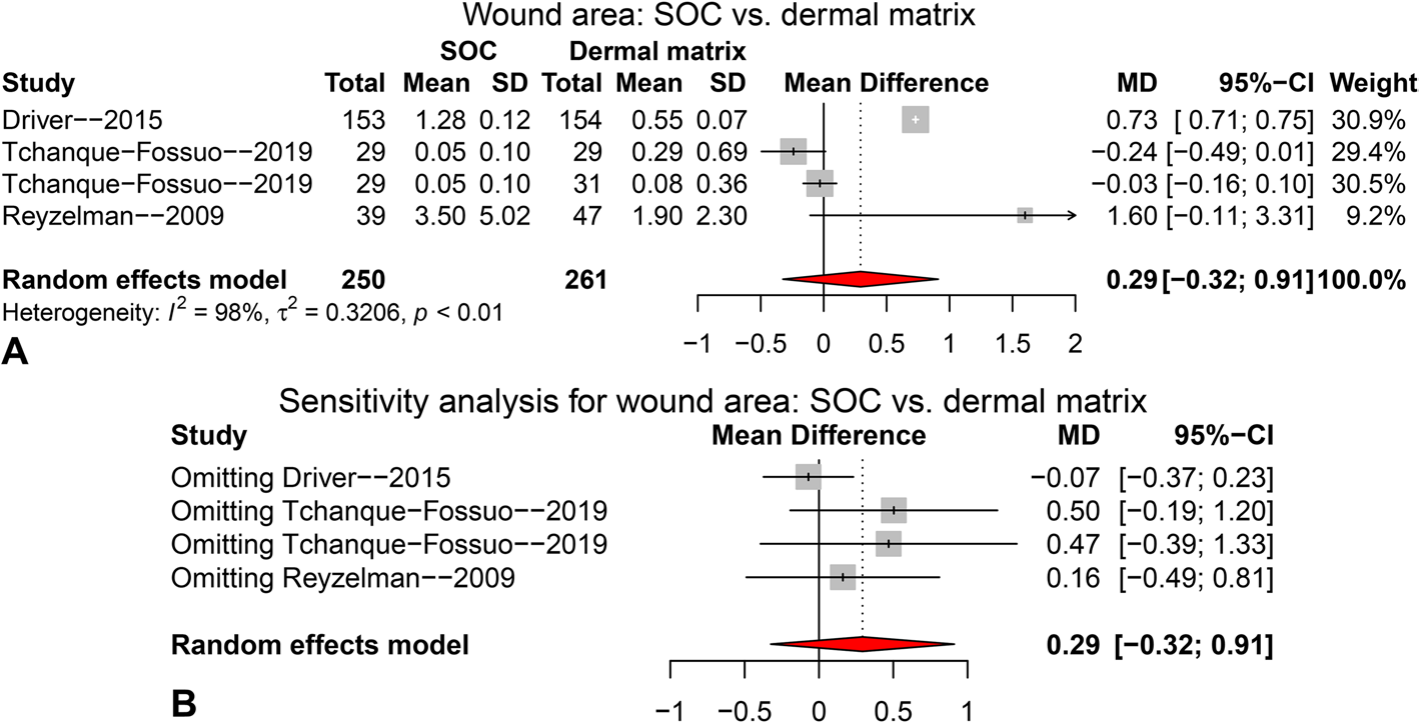
The pooling result for wound area compared between SOC and dermal matrix groups at final follow-up. **A** forest plot; **B** forest plot for sensitivity analysis

Figure 6 shows the comparison of wound area between SOC and dermal matrix, which pooled the data from three studies and four arms using random effects model (I^2^ = 98%). No significant difference between two groups was found (Fig. 6A, MD = 0.29, 95%CI: − 0.32 ~ 0.91, *p* = 0.352). The forest plot of sensitivity analysis (Fig. 6B) showed there was no arm caused significant influence on the pooling result.

### Safety of the dermal matrix in DFU

**Fig. 7:**
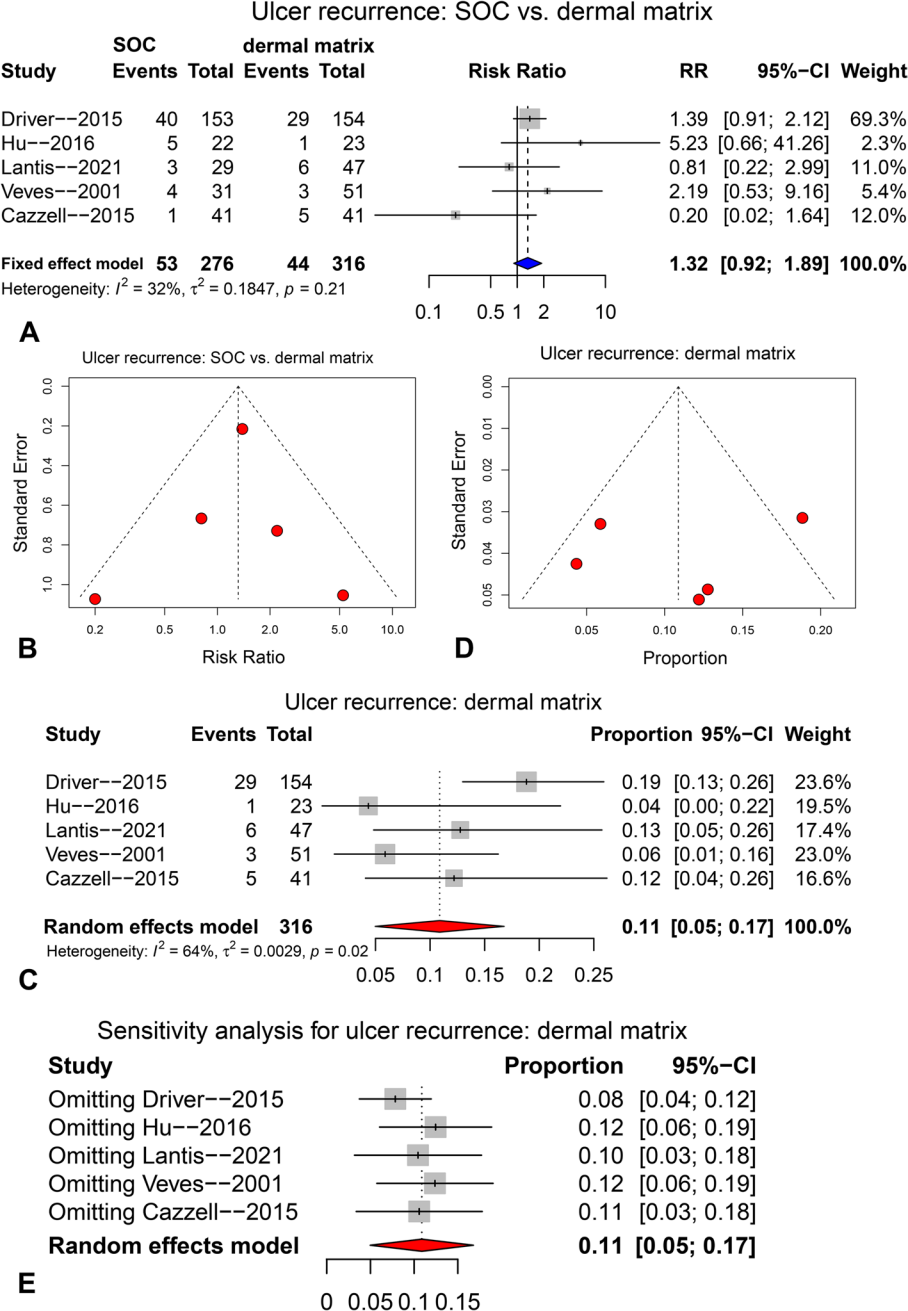
The pooling results for ulcer recurrence rate compared between SOC and dermal matrix groups (**A** and **B**), and for ulcer recurrence rate of dermal matrix group (**C**-**E**), at final follow-up. **A** & **C**. forest plot; **B** & **D***. funnel* plot; **E** forest plot for sensitivity analysis

The ulcer recurrence rate comparison between SOC and dermal matrix is presented in Fig. 7A-B. Five studies were pooled with fixed-effect model (I^2^ = 32%), and no significant difference in ulcer recurrence rate was observed between the two groups (Fig. 7A, RR = 1.32, 95%CI: 0.92 ~ 1.89, *p* = 0.138). The funnel plot (Fig. 7B), Egger’s test (*p* = 0.827) and Begg’s test (*p* = 1.000) did not show any significant publication bias. Sensitivity analysis was not conducted as there was no significant heterogeneity.

The pooling result for ulcer recurrence rate of dermal matrix group at final follow-up is shown in Fig. 7C-E. Five studies were pooled with random-effect model (I^2^ = 64%), resulting in a pooled ulcer recurrence rate of 0.11 (95CI: 0.05 ~ 0.17) for the dermal matrix group (see the forest plot in Fig. 7C). The forest plot of sensitivity analysis (Fig. 7E) showed that no study had a significant influence on the pooled result. The funnel plot (Fig. 7D), Egger’s test (*p* = 0.738) and Begg’s test (*p* = 1.000) did not indicate the presence of significant publication bias.

**Fig. 8:**
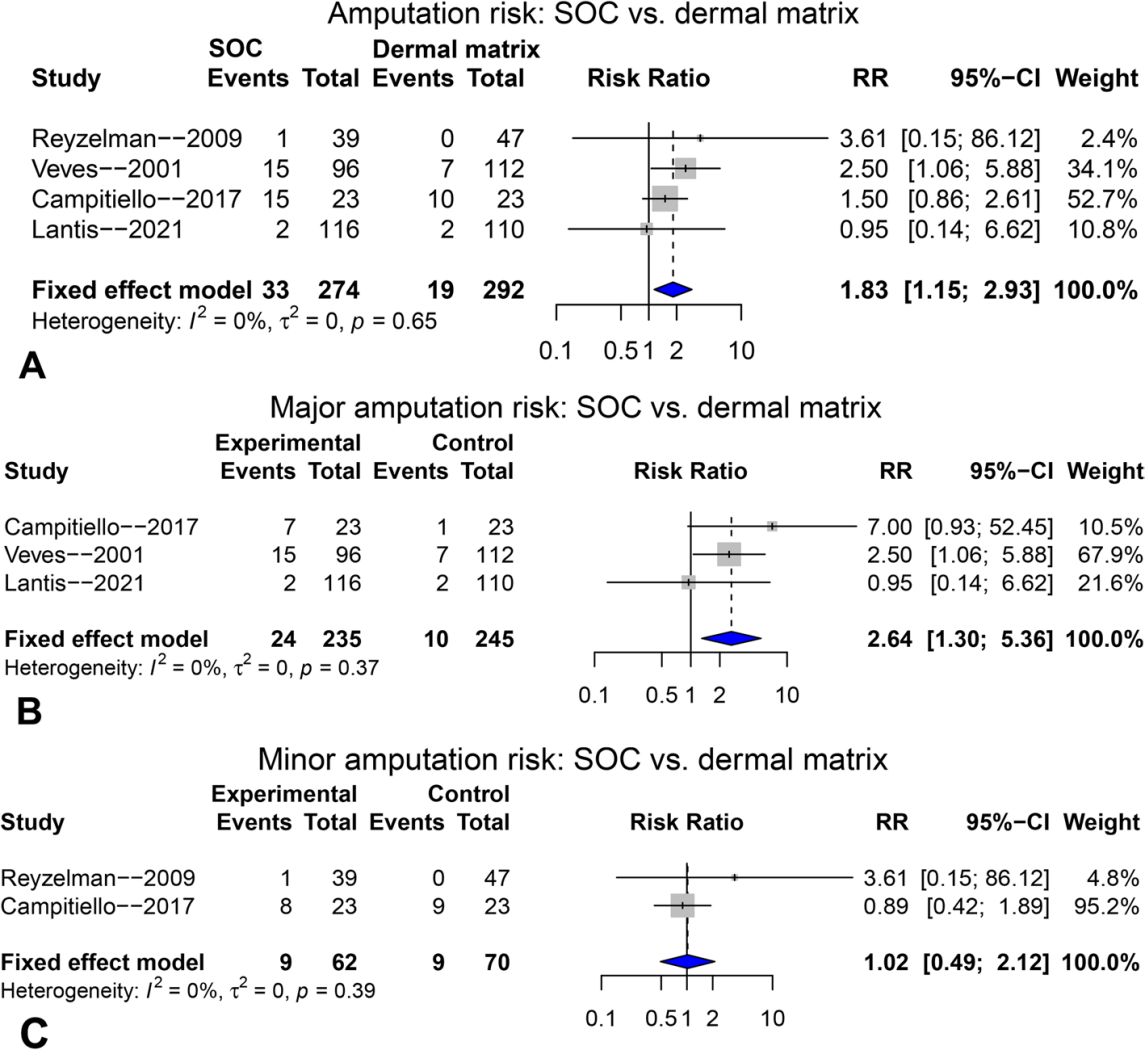
The pooling results for overall amputation risk (**A**) major amputation risk (**B**) and minor amputation risk (**C**) compared between SOC and dermal matrix groups, at final follow-up

Figure 8A shows the forest plot comparing the overall amputation risk between SOC and dermal matrix groups using fixed-effect model (I^2^ = 0%), demonstrating that dermal matrix application could significantly lower the overall amputation risk (RR = 1.83, 95%CI: 1.15 ~ 2.93, *p* = 0.011*). After then, subgroup analyses were conducted for both major (Fig. 8B) and minor (Fig. 8C) amputation risks, showing that dermal matrix application could significantly lower the major amputation risk (RR = 2.64, 95%CI: 1.30 ~ 5.36, *p* = 0.007**), but had no significant impact on the minor amputation risk (RR = 1.02, 95%CI: 0.49 ~ 2.12, *p* = 0.959). Publication bias test and sensitivity analysis were not performed.

**Fig. 9:**
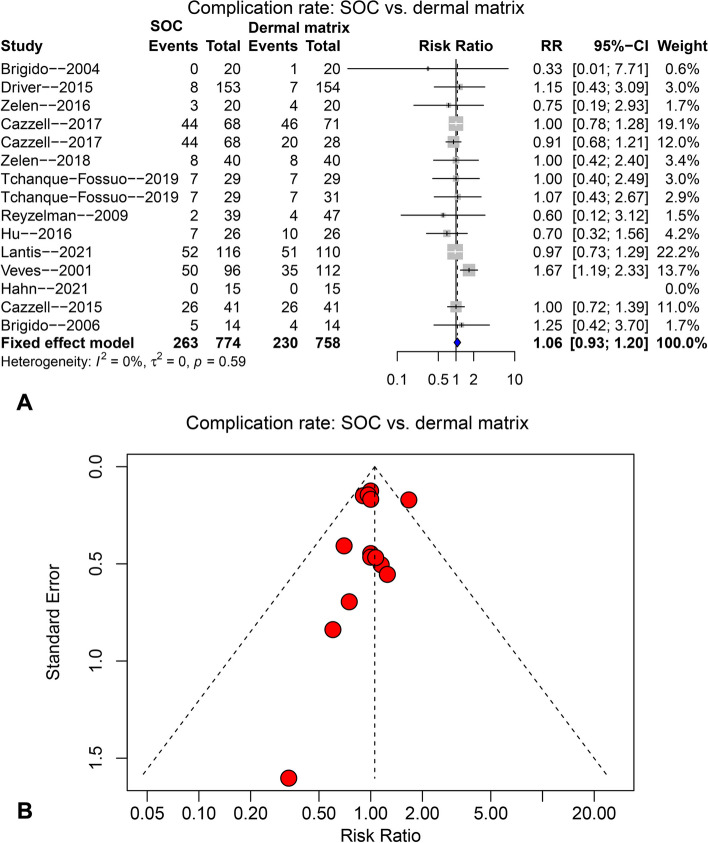
The pooling result for complication risk compared between SOC and dermal matrix groups, at final follow-up. **A** forest plot; **B** funnel plot

The comparison of complication rate between SOC and dermal matrix is presented in Fig. 9. Thirteen studies and 15 arms were pooled with fixed-effect model (I^2^ = 0%), and no significantly different complication rate was observed between two groups (Fig. 9A, RR = 1.06, 95%CI: 0.93 ~ 1.20, *p* = 0.409). The funnel plot (Fig. 9B), Egger’s test (*p* = 0.494) and Begg’s test (*p* = 0.622) did not indicate the presence of significant publication bias. Sensitivity analysis was not conducted as there was no significant heterogeneity.

## Discussion

In this study, we performed a systematic review and meta-analysis based on high-level evidence from RCTs, and found that application of dermal matrix was associated with significantly shorter time to complete healing, increased healing rate, and reduced amputation risk, compared to SOC alone. However, there was no significant difference in wound area, ulcer recurrence and complication risk, between the two groups.

DFUs are always characterized by chronicity and recurrence, making them difficult to fully heal and potentially leading to minor or major limb amputations. The clinical challenges related with DFUs treatment have spawned multiple adjuvant techniques to improve the wound healing. Usually, an ulcer continued for more than 4 weeks is qualified as chronic wound, which can present additional challenges to complete the wound healing because of infection, biofilm formation, and underlying tissue desiccation that cause exacerbated conditions and disturbed healing process. Multiple biologic dressings have been applied in clinical researches in the setting of DFUs, showing promise treatment outcomes. Martin et al. [37] evaluated the outcomes of 17 consecutive patients with neuropathic diabetic foot wounds treated with an acellular matrix, which showed a 20-week healing rate of 82.4% with an average healing time of 8.9 ± 2.7 weeks. Lee et al. [38] compared the efficacy of applying a paste formulation of acellular dermal matrix (ADM) with conventional foam dressing in treating DFUs, reporting an increased healing rate (56.52% vs. 23.08%), increased ratio of healed area (74.17% ± 30.84% vs. 51.87% ± 32.81%) and deceased length of time to heal (13.54 ± 9.18 vs. 21.5 ± 11.98 days) in ADM group, at the 60-day primary outcome mark. Zelen et al. [25] compared the clinical outcomes of human reticular acellular dermis matrix (HR-ADM) versus SOC to facilitate wound closure in non-healing DFUs. At the final follow-up (12 weeks), DFUs of HR-ADM and SOC groups healed in 80 and 20% of the patients, with a mean healing time of 40 days and 77 days, respectively. There was no significantly increased adverse or serious adverse events between the two groups or any adverse events related to the graft. In another RCT by Hahn et al. [33], the clinical outcomes of a micronized dermal matrix (MDM) was compared with conventional negative-pressure wound therapy (NPWT) in the treatment of DFUs. As a result, all wounds treated with MDM showed healthy granulation tissue without noticeable complications during follow-up. The MDM group showed a higher healing rate compared to NPWT group, at 42 and 120 days, while similar healing rates were achieved between two groups at 6-month follow-up period. In 2017, a systematic review and meta-analysis conducted by Guo et al. [39] compared the efficacy and safety of ADM in DFU treatment, which showed that compared with the SOC alone, the ADM group was associated with higher complete healing rates at 12 and 16 weeks, and shorter mean time to complete wound healing. The adverse event rates in both groups were similar, indicating that the use of ADM did not increase the risk of adverse events.

In the current study, we included 15 RCTs involving 1524 patients, and demonstrated that dermal matrix is an effective and safe treatment option for enhancing DFU healing. The results of this study support previous studies documenting the successful application of dermal matrix therapy. Dermal matrix acts as a sterile tissue graft which can be applied directly to wound beds of DFUs and integrate with the surrounding host tissues to actively stimulate cell migration, angiogenesis, and epithelialization, resulting in accelerated wound healing [40]. Although the final follow-up wound area was similar between two treatment groups, the percentage area reduction (PAR) was demonstrated to be significantly increased in several studies [23, 26, 27, 31, 34]. However, the PAR was not pooled by meta-analysis due to the non-availability of the primary data (mean value and standard deviation of PAR).

This study, nevertheless, has several limitations that should be pointed out. Firstly, the dermal matrix products used in the studies varied among different manufacturers, which may introduce potential risk of bias. Secondly, due to the inconcealability of the treatment process with dermal matrix in the primary trials, an additional risk of bias may be caused by the unblinded application of dermal matrix to patients. Additionally, the studies reported outcomes at different follow-up times, making it difficult to pool the data. In this study, we selected the data at the final follow-up to conduct the analyses. Finally, most of the current available RCTs have relatively small sample sizes and short-term follow-up periods, which indicates that some more trials with larger sample size and longer follow-up period are required to provide some more convincing evidence.

## Conclusions

The results of the current meta-analysis demonstrated that the application of dermal matrix as an adjuvant therapy to SOC can effectively enhance the healing process of DFUs and reduce the amputation risk when compared to SOC alone. Additionally, dermal matrix application was well tolerated by the subjects without added complication risk. However, some further well-designed prospective trials with larger sample sizes and longer follow-up periods are required to provide more convincing evidence.

### Supplementary Information

**Additional file 1. **Checklist of the PRISMA for network meta-analysis.

## Data Availability

The datasets used and/or analyzed during the current study are available from the corresponding author on reasonable request.

## Abbreviations

SOC: Standard of care
IDRT: Integra dermal regeneration template
NA: Not available
ADM: Acellular dermal matrix
CDM: Cellular dermal matrix
D-ADM: DermACELL acellular dermal matrix
GJ-ADM: GraftJacket acellular dermal matrix
Pre-D: Prediabetes
STSG: Split-thickness skin grafting
NPWT: Negative-pressure wound therapy
SIS: Small intestine submucosa
BMI: Body mass index
DFU: Diabetic foot ulcer
DFI: Diabetic foot infection
RCTs: Randomized controlled trials
MD: Mean difference
OR: Odds ratio
RR: Risk ratio
QoF: Quality of life
PRISMA: Preferred reporting items for systematic reviews and meta-analyses
DPN: Diabetic polyneuropathy
PP: Per-protocol
ITT: Intention-to-treat
95% CI: 95% Confidence interval
HR-ADM: Human reticular acellular dermis matrix
MDM: Micronized dermal matrix

## References

[1] Armstrong DG, Boulton AJM, Bus SA (2017). Diabetic foot ulcers and their recurrence. N Engl J Med.

[2] Nabuurs-Franssen MH, Huijberts MS, Nieuwenhuijzen Kruseman AC, Willems J, Schaper NC (2005). Health-related quality of life of diabetic foot ulcer patients and their caregivers. Diabetologia..

[3] Ragnarson Tennvall G, Apelqvist J (2000). Health-related quality of life in patients with diabetes mellitus and foot ulcers. J Diabetes Complicat.

[4] Armstrong DG, Lavery LA, Harkless LB (1998). Validation of a diabetic wound classification system: the contribution of depth, infection, and ischemia to risk of amputation. Diabetes Care.

[5] Oyibo SO, Jude EB, Tarawneh I, Nguyen HC, Harkless LB, Boulton AJM (2001). A comparison of two diabetic foot ulcer classification systems: the Wagner and the University of Texas wound classification systems. Diabetes Care.

[6] Santema TB, Poyck PP, Ubbink DT (2016). Skin grafting and tissue replacement for treating foot ulcers in people with diabetes. Cochrane Database Syst Rev.

[7] Singer AJ, Tassiopoulos A, Kirsner RS (2017). Evaluation and Management of Lower-Extremity Ulcers. N Engl J Med.

[8] Driver VR, Fabbi M, Lavery LA, Gibbons G (2010). The costs of diabetic foot: the economic case for the limb salvage team. J Vasc Surg.

[9] Boulton AJ, Vileikyte L, Ragnarson-Tennvall G, Apelqvist J (2005). The global burden of diabetic foot disease. Lancet..

[10] Lev-Tov H, Li CS, Dahle S, Isseroff RR (2013). Cellular versus acellular matrix devices in treatment of diabetic foot ulcers: study protocol for a comparative efficacy randomized controlled trial. Trials..

[11] Everett E, Mathioudakis N (2018). Update on management of diabetic foot ulcers. Ann N Y Acad Sci.

[12] Margolis DJ, Allen-Taylor L, Hoffstad O, Berlin JA (2005). Healing diabetic neuropathic foot ulcers: are we getting better?. Diabet Med.

[13] Aldana PC, Khachemoune A (2020). Diabetic foot ulcers: appraising standard of care and reviewing new trends in management. Am J Clin Dermatol.

[14] Snyder D, Sullivan N, Margolis D, Schoelles K (2020). Skin substitutes for treating chronic wounds [internet].

[15] Climov M, Bayer LR, Moscoso AV, Matsumine H, Orgill DP (2016). The role of dermal matrices in treating inflammatory and diabetic wounds. Plast Reconstr Surg.

[16] Cho H, Blatchley MR, Duh EJ, Gerecht S (2019). Acellular and cellular approaches to improve diabetic wound healing. Adv Drug Deliv Rev.

[17] Reyzelman AM, Bazarov I (2015). Human acellular dermal wound matrix for treatment of DFU: literature review and analysis. J Wound Care.

[18] Deng W, Boey J, Chen B (2016). Platelet-rich plasma, bilayered acellular matrix grafting and negative pressure wound therapy in diabetic foot infection. J Wound Care.

[19] Luthringer M, Mukherjee T, Arguello-Angarita M, Granick MS, Alvarez OM (2020). Human-derived acellular dermal matrix grafts for treatment of diabetic foot ulcers: a systematic review and Meta-analysis. Wounds..

[20] Moher D, Liberati A, Tetzlaff J, Altman DG, PRISMA Group (2009). Preferred reporting items for systematic reviews and meta-analyses: the PRISMA statement. BMJ..

[21] Higgins JP, Altman DG, Gøtzsche PC (2011). The Cochrane Collaboration’s tool for assessing risk of bias in randomised trials. BMJ..

[22] Brigido SA, Boc SF, Lopez RC (2004). Effective management of major lower extremity wounds using an acellular regenerative tissue matrix: a pilot study. Orthopedics..

[23] Driver VR, Lavery LA, Reyzelman AM (2015). A clinical trial of Integra template for diabetic foot ulcer treatment. Wound Repair Regen.

[24] Cazzell S, Moyer PM, Samsell B, Dorsch K, McLean J, Moore MA (2019). A prospective, multicenter, single-arm clinical trial for treatment of complex diabetic foot ulcers with deep exposure using acellular dermal matrix. Adv Skin Wound Care.

[25] Zelen CM, Orgill DP, Serena T (2017). A prospective, randomised, controlled, multicentre clinical trial examining healing rates, safety and cost to closure of an acellular reticular allogenic human dermis versus standard of care in the treatment of chronic diabetic foot ulcers. Int Wound J.

[26] Cazzell S, Vayser D, Pham H (2017). A randomized clinical trial of a human acellular dermal matrix demonstrated superior healing rates for chronic diabetic foot ulcers over conventional care and an active acellular dermal matrix comparator. Wound Repair Regen.

[27] Zelen CM, Orgill DP, Serena TE (2018). An aseptically processed, acellular, reticular, allogenic human dermis improves healing in diabetic foot ulcers: a prospective, randomised, controlled, multicentre follow-up trial. Int Wound J.

[28] Tchanque-Fossuo CN, Dahle SE, Lev-Tov H (2019). Cellular versus acellular matrix devices in the treatment of diabetic foot ulcers: interim results of a comparative efficacy randomized controlled trial. J Tissue Eng Regen Med.

[29] Reyzelman A, Crews RT, Moore JC (2009). Clinical effectiveness of an acellular dermal regenerative tissue matrix compared to standard wound management in healing diabetic foot ulcers: a prospective, randomised, multicentre study. Int Wound J.

[30] Hu Z, Zhu J, Cao X (2016). Composite skin grafting with human acellular dermal matrix scaffold for treatment of diabetic foot ulcers: a randomized controlled trial. J Am Coll Surg.

[31] Lantis JC, Snyder R, Reyzelman AM (2021). Fetal bovine acellular dermal matrix for the closure of diabetic foot ulcers: a prospective randomised controlled trial. J Wound Care..

[32] Veves A, Falanga V, Armstrong DG, Sabolinski ML, Apligraf Diabetic Foot Ulcer Study (2001). Graftskin, a human skin equivalent, is effective in the management of noninfected neuropathic diabetic foot ulcers: a prospective randomized multicenter clinical trial. Diabetes Care.

[33] Hahn HM, Lee DH, Lee IJ (2021). Ready-to-use micronized human acellular dermal matrix to accelerate wound healing in diabetic foot ulcers: a prospective randomized pilot study. Adv Skin Wound Care.

[34] Cazzell SM, Lange DL, Dickerson JE, Slade HB (2015). The Management of Diabetic Foot Ulcers with porcine small intestine submucosa tri-layer matrix: a randomized controlled trial. Adv Wound Care (New Rochelle).

[35] Brigido SA (2006). The use of an acellular dermal regenerative tissue matrix in the treatment of lower extremity wounds: a prospective 16-week pilot study. Int Wound J.

[36] Campitiello F, Mancone M, Della Corte A, Guerniero R, Canonico S (2017). To evaluate the efficacy of an acellular Flowable matrix in comparison with a wet dressing for the treatment of patients with diabetic foot ulcers: a randomized clinical trial. Updat Surg.

[37] Martin BR, Sangalang M, Wu S, Armstrong DG (2005). Outcomes of allogenic acellular matrix therapy in treatment of diabetic foot wounds: an initial experience. Int Wound J.

[38] Lee M, Jun D, Choi H, Kim J, Shin D (2020). Clinical efficacy of acellular dermal matrix paste in treating diabetic foot ulcers. Wounds..

[39] Guo X, Mu D, Gao F (2017). Efficacy and safety of acellular dermal matrix in diabetic foot ulcer treatment: a systematic review and meta-analysis. Int J Surg.

[40] Macri L, Clark RA (2009). Tissue engineering for cutaneous wounds: selecting the proper time and space for growth factors, cells and the extracellular matrix. Skin Pharmacol Physiol.

